# Biostack: Nontoxic Metabolite Detection from Live Tissue

**DOI:** 10.1002/advs.202101711

**Published:** 2021-11-05

**Authors:** Xenofon Strakosas, Mary J. Donahue, Adel Hama, Marcel Braendlein, Miriam Huerta, Daniel T. Simon, Magnus Berggren, George G. Malliaras, Roisin M. Owens

**Affiliations:** ^1^ Laboratory of Organic Electronics Department of Science and Technology Linköping University Norrköping 601 74 Sweden; ^2^ King Abdullah University of Science and Technology KAUST Thuwal 23955‐6900 Saudi Arabia; ^3^ Panaxium Aix en Provence 13100 France; ^4^ Robert F. Smith School of Chemical and Biomolecular Engineering Cornell University Ithaca NY 14853 USA; ^5^ Electrical Engineering Division University of Cambridge Cambridge CB3 0FA UK; ^6^ Department of Chemical Engineering and Biotechnology University of Cambridge Cambridge UK USA

**Keywords:** biofunctionalization, biosensors, glucose monitoring, organic bioelectronics, organic electrochemical transistors

## Abstract

There is increasing demand for direct in situ metabolite monitoring from cell cultures and in vivo using implantable devices. Electrochemical biosensors are commonly preferred due to their low‐cost, high sensitivity, and low complexity. Metabolite detection, however, in cultured cells or sensitive tissue is rarely shown. Commonly, glucose sensing occurs indirectly by measuring the concentration of hydrogen peroxide, which is a by‐product of the conversion of glucose by glucose oxidase. However, continuous production of hydrogen peroxide in cell media with high glucose is toxic to adjacent cells or tissue. This challenge is overcome through a novel, stacked enzyme configuration. A primary enzyme is used to provide analyte sensitivity, along with a secondary enzyme which converts H_2_O_2_ back to O_2_. The secondary enzyme is functionalized as the outermost layer of the device. Thus, production of H_2_O_2_ remains local to the sensor and its concentration in the extracellular environment does not increase. This “biostack” is integrated with organic electrochemical transistors to demonstrate sensors that monitor glucose concentration in cell cultures in situ. The “biostack” renders the sensors nontoxic for cells and provides highly sensitive and stable detection of metabolites.

## Graphical abstract

A “biostack” functionalization method is demonstrated for transistor‐based metabolite sensors. This technique makes use of a double enzyme stack which limits hydrogen peroxide accumulation in biological media. As a result real time glucose measurement is possible in the presence of cells.

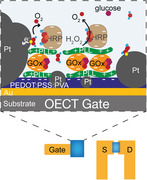

## 1 Introduction

Although secreted metabolites may be monitored using offline biosensors, there is increasing demand for direct in situ metabolite monitoring from live cell cultures as well as in vivo with implanted devices. Applications include monitoring of metabolites such as glucose and lactate, key components for assessing metabolism in cells and tissues, and important indicators for diseases such as epilepsy, diabetes, and cancer.^[^ ^1^, ^2^ ^]^ For example, glucose is a well‐known biomarker in the study and diagnosis of diabetes. During an epileptic crisis there is an increased consumption of glucose and increased secretion of lactate. ^[^ ^3^, ^4^ ^]^ Additionally, drugs or a pathogen may alter the metabolism of a cell, and in vitro drug testing platforms integrated with live cells have been developed for toxicology tests.^[^ ^5^ ^]^

Biosensors represent a useful technology to understand and diagnose diseases. An effective biosensor must have high sensitivity and high specificity. A host of novel technologies have emerged with sensitivities into the attomolar range,^[^ ^6^, ^7^ ^]^ however, achieving specificity relies on having a good biorecognition element (BRE). Nature's solution is to use proteins to achieve specificity, easily distinguishing between pairs of enantiomers. Incorporation of proteins into biosensors as BREs can be very effective, however, if the sensor must operate in vivo, or even in vitro (with live cells), a variety of problems may arise. Many metabolite sensors use redox enzymes as the BRE with an operating principle based on the Clark electrode.^[^ ^8^ ^]^ An electrode, usually platinum, measures the faradaic current from the decomposition of hydrogen peroxide (H_2_O_2_), which is produced as a by‐product when the relevant metabolites are converted by their oxidase enzymes. The most advanced electrochemical sensors, called second and third generation sensors, employ electrochemically active species bound to polymers for the transfer of electrons to the transducer. However, the mediators utilized for these sensors are often cytotoxic.^[^ ^9^ ^]^ Commercial blood glucose and lactate sensors are available, but typically necessitate the extraction of a sample (blood, serum, saliva), prior to measurement. Although there has been a push toward continuous monitoring of metabolites,^[^ ^10^ ^]^ challenges have prohibited their widespread success. These challenges include dual hurdles of low stability and poor biocompatibility, not only of the transducer but also of the BRE or its byproducts, and background noise from electroactive species.

An effective strategy for in vivo*/*in vitro detection of metabolites should thus be specific, sensitive, and capable of operating in complex matrices such as biological fluids and tissues. For implantable use, mechanical compliance is also required to avoid extensive immune reaction.^[^ ^11^ ^]^ Finally, it is exceedingly important that the device is stable and biocompatible and that there is no toxicity either from either the transducer or the BRE and its byproducts. Organic electronics have emerged as alternatives to silicon‐based electronics used to integrate with live cells and tissues, primarily due to their tissue‐like mechanical properties and their mixed ionic/electronic conduction.^[^ ^12^, ^13^ ^]^ Among emerging organic electronic devices, the organic electrochemical transistor (OECT) has been used extensively for interfacing with biological systems,^[^ ^14^ ^]^ and has shown excellent biocompatibility with live tissues both in vitro^[^ ^15^, ^16^ ^]^ and in vivo.^[^ ^17^ ^]^ The OECT displays enhanced signal transduction of biological signals^[^ ^17^ ^]^ and metabolite sensing using the OECT has been demonstrated repeatedly through coupling of enzymes with and without mediators.^[^ ^18^, ^19^, ^20^, ^21^ ^]^ In some cases incorporation of materials such as platinum nanoparticles (Pt‐NPs) were used to enhance catalytic activity have reached sensitivities at the nanomolar range for the detection of glucose and dopamine.^[^ ^22^, ^23^ ^]^ A significant advantage of organic electronic devices such as the OECT is their amenability to chemical modification or functionalization.^[^ ^24^ ^]^ Stability of metabolite sensing with OECTs has been improved by the covalent immobilization of enzymes in close proximity to the active electrode.^[^ ^20^, ^25^, ^26^ ^]^ Owing to their operating principle, however, such redox enzyme sensors are incompatible with operation in direct contact with cells and tissue, both due to toxicity of electrochemically active mediators that can leach out of the device or via the production of H_2_O_2_, which is highly toxic to the cells. When H_2_O_2_ is produced as the byproduct of a redox reaction in the presence of oxygen, a certain proportion will directly transfer electrons to the transducer; however, a significant amount may also diffuse into the biological milieu and thus affect the integrity of the tissue. This results in a limited utilization of current enzymatic sensors with live cells.

Here, we report an OECT‐based metabolite sensor that is capable of continuously monitoring relevant metabolites in close proximity to cells and tissue, without disrupting their normal function. This is possible through an approach termed here the “biostack,” in which a layered functionalization approach is used to place the redox enzyme corresponding to the metabolite of interest as close as possible to the electrode, while the upper layer consists of horseradish peroxidase (HRP) or catalase (Cat) that neutralizes the diffusing H_2_O_2_. The active enzymes are separated by polypeptide spacers that enhance and stabilize the interactions. The inclusion of Pt‐NPs improves catalytic efficiency, and additionally provides a high surface area for charge transfer reactions to take place as a result of the 3D structure at the gate electrode (Figure S1, Supporting Information). This simple but effective stacking approach not only demonstrates sensitivities tuned to the application at hand, but it also maintains high stabilities in complex in vitro situations, inhibiting toxicity to cells cultivated adjacent to the electrodes.

## 2 Results

**Figure 1:**
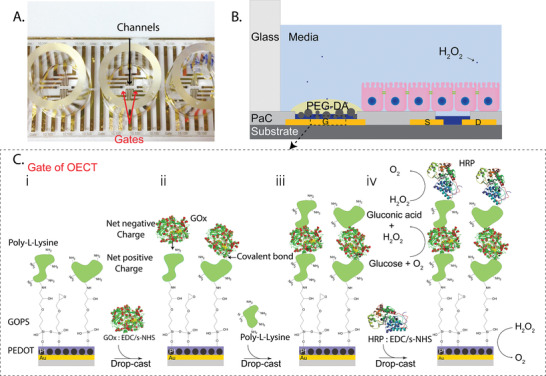
Design and fabrication of the OECT within the biostack platform. A) Photograph of the device showing the glass wells, gate electrodes, and channels. Well diameter is 8 mm. B) Schematic showing a cross‐section of a portion of the device contained within a glass well, one gate, and one OECT, integrated with cells. The gates (functionalized and control) of the OECTs are covered with a PEG‐DA gel in order to prevent cells from adhering to the gates. C) Functionalization scheme for the biostack on top of the gate electrodes. An epoxy silane (GOPS) covalently binds to PEDOT:PSS:PVA and poly‐l‐lysine (PLL) binds to the epoxy group of the GOPS. Modified glucose oxidase (GOx) with EDC/s‐NHS binds to the amine groups of PLL. The PLL deposition and enzyme activation processes are repeated to immobilize a peroxidase enzyme such as HRP or Cat in a stack configuration on top of GOx or LOx.

The metabolite sensors developed here are based on OECT devices created using previously reported fabrication techniques on fused silica glass slides.^[^ ^27^ ^]^ The design allows for the integration of four glass wells on each glass substrate, used to confine the electrolyte. In each well there are two planar electrodes used as gates for the transistors as well as six channels for testing and measurement as depicted in **Figure** **1**A. Our goal was to integrate the OECT‐based sensors with cells to monitor their metabolic activities in vitro and in real time (Figure 1B). The cultured cells create a confluent layer on top of planar OECT sensors, apart from the gate on which a polyethylene glycol diacrylate (PEG‐DA) gel was drop‐cast and crosslinked by UV radiation, to allow diffusion of metabolites to the gate. In order to monitor metabolites in vitro, it is necessary to reduce the inherent toxicity of first‐generation enzymatic metabolite sensors, which results from the production and out‐diffusion of H_2_O_2_.^[^ ^16^ ^]^ In the devices developed here, the functionalization and sensing occurs at the gate electrode, whereas the channel transduces the signal to an electronic current that can be monitored without the need of external amplification.^[^ ^28^ ^]^ A blend of PEDOT:PSS (poly(3,4‐ethylenedioxythiophene) polystyrene sulfonate) with polyvinyl alcohol (PVA) was used^[^ ^25^ ^]^ to enable the further chemical functionalization of the gate electrode according to the scheme in Figure 1C. The functionalization consists of the “biostack” approach utilizing sequential deposition of Pt‐NPs, an epoxy silane (GOPs), poly‐l‐lysine (PLL), glucose oxidase (GOx) or lactate oxidase (LOx), PLL, and lastly HRP or Cat. Post device fabrication and prior to “biostack” functionalization, the Pt‐NPs were introduced into the conducting polymer film at the gate by electrodeposition.^[^ ^26^, ^29^, ^30^ ^]^ As the reduction potential is only applied at the gate and not the OECT channels, the Pt‐NPs were deposited only at the gate electrode. Pt was used because of its excellent catalytic activity in the decomposition of H_2_O_2_, with an additional advantage coming from the high surface area generated,^[^ ^31^ ^]^ facilitating an increased and more rapid breakdown of H_2_O_2_ (Figure S1, Supporting Information). After Pt‐NP deposition, a monolayer of a heterobifunctional silane (3‐glycidoxy‐propyltrimethoxysilane (GOPS)) was deposited via chemical vapor deposition (CVD), relying on the binding of silanol groups to the alcohol group of PVA. This monolayer binding leaves epoxy groups available for subsequent coupling to the amine groups in PLL. PLL was drop cast on top of the gate, left for 2 h, and then washed thoroughly with phosphate buffer saline (PBS) and deionized (DI) water (Figure 1C i ). The addition of PLL serves two purposes: It provides a more attractive surface for subsequent adsorption of enzymes GOx or LOx (with isoelectric points of 4.2 and 4.6 respectively, thus negatively charged at physiological pH) than bare PEDOT:PSS, which is assumed to have a significant negative charge near the surface due to excess PSS. The positively charged PLL thus neutralizes the repulsive effects between the negatively charged PSS and subsequently attracts the negatively charged GOx/LOx, resulting in a high loading of enzyme on the surface (Figure S3A–C, Supporting Information). Owing to the high molecular weight and linear structure (and therefore length) of PLL, it can act as an effective spacer between the enzymatic layers. Prior to deposition, either GOx or LOx (as indicated) were activated by EDC/s‐NHS (1‐ethyl‐3‐(3‐dimethylaminopropyl)carbodiimide/*N*‐hydroxysulfosuccinimide) chemistry, in order to assist in formation of covalent bonds with the underlying PLL (Figure 1C ii). ^[^ ^32^, ^33^ ^]^

**Figure 2:**
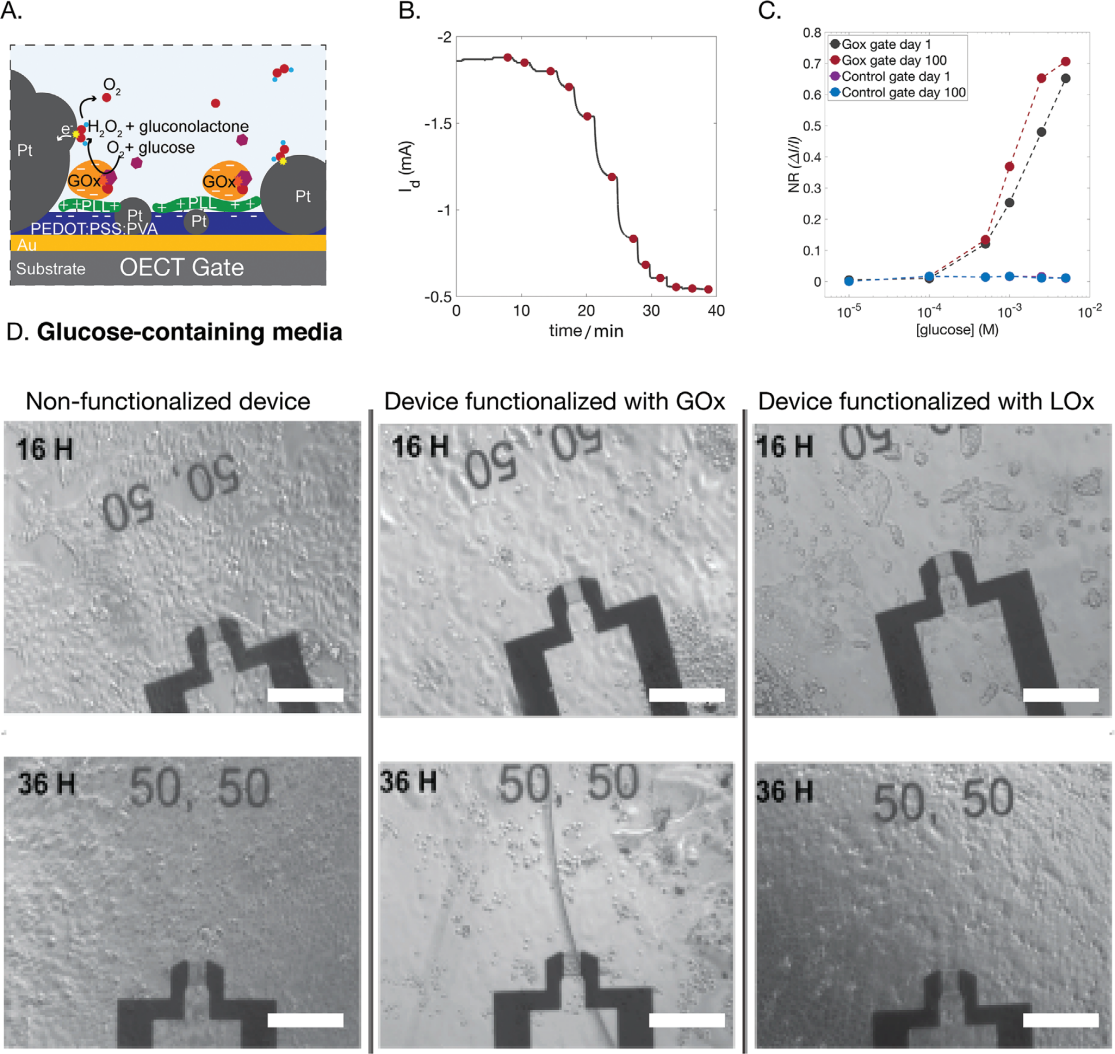
Functionalization of OECTs with redox enzymes results in a device with high sensitivity but significant cytotoxicity due to production of hydrogen peroxide (H_2_O_2_). A) Schematic of a device functionalized with GOx, on top of PLL and in close proximity to Pt‐NPs. B) Typical current response of a GOx functionalized device with a constant *V*_g_ = 0.4 V upon increasing concentrations of glucose in the electrolyte (the red points indicate addition steps from 1 × 10^−6^ to 5 × 10^−3^ m with the concentration doubling at each step). C) Normalized current response between a freshly functionalized and an OECT stored in PBS at 4 °C for 100 d. D) Toxicity of the OECT glucose sensor, integrated with an MDCK II epithelial cell line. The cells grow to form a confluent layer on nonfunctionalized OECTs after 36 h, left panel (top 16 h after seeding the cell, bottom 36 h after seeding the cells). Cells do not grow on GOx functionalized OECT middle panel. Slower cell growth is observed on an LOx functionalized OECT right panel. The toxicity is attributed to the production of H_2_O_2_ in cell media containing 5 × 10^−3^ m of glucose. Scale bars: 200 µm.

Our strategy was designed to covalently immobilize the GOx/LOx as close as possible to the gate electrode, ensuring that when glucose is converted to H_2_O_2_ it can diffuse rapidly to the Pt‐NP decorated gate electrode, resulting in an increased sensing response (**Figure** **2**A) as well as long‐term high stability. In the presence of O_2_, glucose is converted to gluconolactone by the enzyme glucose oxidase and lactate is converted to pyruvate by lactate oxidase, with H_2_O_2_ as a byproduct of the coupled reaction. H_2_O_2_ in turn is catalyzed by the Pt‐NPs, giving two electrons to the gate when a constant positive *V*_g_ is applied.^[^ ^34^ ^]^ This electron transfer process results in dedoping of the PEDOT channel and thus modulation of the source–drain current (*I*_d_). The modulation is proportional to the concentration of produced H_2_O_2_ and thus of the analyte (glucose, lactate).^[^ ^34^ ^]^ Figure 2B shows a representative curve of the measured current response, when glucose was added to the system in a range of concentrations from 1 × 10^−6^ to 5 × 10^−3^ m. The current modulation was reproducible and depended on the concentration of the analyte for a range of concentrations when initial high concentration of glucose was depleted or when glucose was added with washing steps in between, in which the electrolyte was replaced with a nonglucose buffer (Figure S2A–C, Supporting Information). We believe the observed stability of the sensors over time is a result of covalent binding of GOx to the substrate. Figure 2C shows the normalized response calibration curves and control gates, extracted from a glucose sensor prior to (red) and after (black) storage in PBS at 4 °C for 100 d. The sensor is functional in the same glucose concentration range. This functionality after storage in PBS in Figure 2C, as well as the measurements in Figure S2A,B in the Supporting Information, indicate a covalent arrangement, and likely covalent bonding between the GOx/PLL/GOPS/PEDOT:PSS:PVA layers. Evidence for an increased loading of the GOx enzyme through the addition of PLL is given by quartz crystal microbalance (QCM) data (Figure S3A,B, Supporting Information). QCM frequency changes are proportional to the amount of adsorbed mass on a surface. In the presence of immobilized PLL, the QCM frequency change during introduction of GOx is larger, and it remains larger after a washing step, compared to when PLL is not pre‐immobilized on PEDOT:PSS:PVA (Figure S3C, Supporting Information). The higher amount of immobilized enzyme resulted in an enhanced sensitivity and stability of operation of the device (Figure S3D, Supporting Information).

Since H_2_O_2_ is neutral, only a portion of it is converted at the Pt‐NPs gate electrode and the remainder diffuses out from the surface to the biological media. If such sensors are to be used in proximity to live cells or tissue, the H_2_O_2_ must be removed. Indeed, experiments carried out cultivating live cells on the OECT glucose sensors clearly show that the cells experience significant toxicity on substrates with GOx functionalized electrodes (Figure 2D) compared to nonfunctionalized devices or LOx functionalized electrodes. Madin Darby canine kidney (MDCK II) epithelial cells (canine kidney epithelial cells) cultivated using media containing galactose as an alternative carbon source, on devices with GOx functionalized electrodes, showed no evident toxicity, confirming the role of the glucose enzyme in the toxicity effects (Figure S4A, Supporting Information). In addition, there is the appearance of slower growth of cells on LOx functionalized devices at 16 h, but they are fully recovered at 36 h. This indicates some production of lactate from the cells (there is no lactate in the media), which is converted to H_2_O_2_ at the gate electrode, which is toxic to the cells. Further experiments cultivating cells in the presence of media collected from the GOx functionalized devices, but on tissue culture dishes, also showed cells with rounded morphology and some areas where no cells were grown (Figure S4B, Supporting Information, black arrows). The changes in the morphology of MDCK cells are a known sign of cell damage.^[^ ^35^, ^36^ ^]^ This implies the presence of a soluble toxic compound in the media (presumably H_2_O_2_).

**Figure 3:**
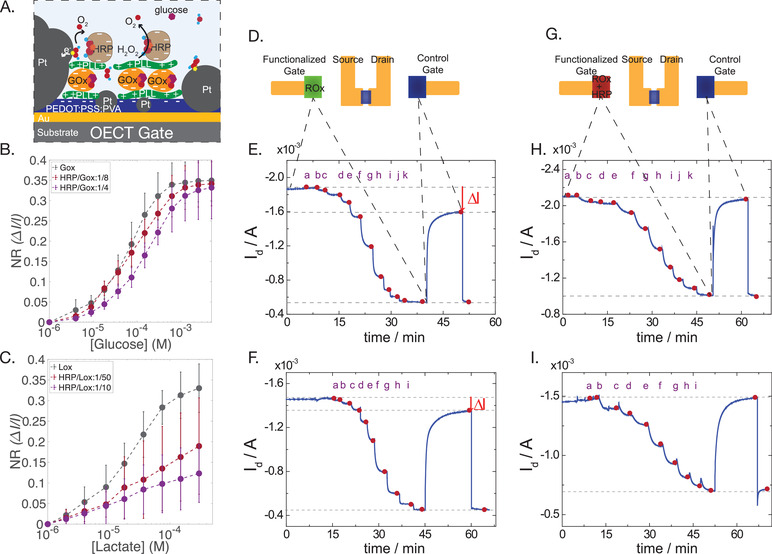
Biostack device functionalized with both redox and peroxidase enzymes. A. Schematic of a device with the addition of second PLL layer and HRP. B. Binding of HRP on a GOx functionalized gate shifts the glucose detection range to higher concentrations (*n* = 3 gates for each condition and two time series measurements for each gate). C) Binding of HRP to an LOx functionalized gate shifts and reduces the sensitivity (*n* = 3 gates for each condition and two time series measurements for each gate). D) Illustration of a device operated using two gates: first the OECT is modulated using the gate functionalized with GOx or LOx (ROx) and secondly using a control gate with only PtNPs. Both gates are located in the same electrolyte. E) Typical current response of GOx functionalized device *without the secondary HRP enzyme layer* upon increasing concentrations of glucose in the electrolyte (*a* = 0 × 10^−6^ m, *b* = 5 × 10^−6^ m, *c* = 10 × 10^−6^ m, *d* = 20 × 10^−6^ m, *e* = 50 × 10^−6^ m, *f* = 100 × 10^−6^ m, *g* = 250 × 10^−6^ m, *h* = 500 × 10^−3^ m, *i* = 1 × 10^−3^ m, *j* = 2.5 × 10^−3^ m, *k* = 5 × 10^−3^ m). When the gate is switched to the control gate, a shift in the baseline between functionalized and control gate is observed. This shift is attributed to diffused H_2_O_2_, produced by the catalysis of glucose at the functionalized gate. F) Analogous to (E), the current response of an LOx functionalized device *without the secondary HRP enzyme layer* upon increasing concentrations of lactate in the electrolyte (*a* = 0 × 10^−6^ m, *b* = 1 × 10^−6^ m, *c* = 5 × 10^−6^ m, *d* = 10 × 10^−6^ m, *e* = 20 × 10^−6^ m, *f* = 50 × 10^−6^ m, *g* = 100 × 10^−6^ m, *h* = 250 × 10^−6^ m, *i* = 500 × 10^−6^ m). A similar shift is observed in the baseline, but smaller due to the smaller lactate concentration. G) Illustration of device using one gate functionalized with GOx/LOx and HRP and one control device with Pt‐NPs. H) Similar to (E), addition of the same glucose concentrations shows sensitivity using the functionalized gate. When changing the OECT gate to the control gate, no shift in the baseline is observed between control and functionalized gate. HRP is functionalized in the stack configuration therefore prevents or significantly reduces H_2_O_2_ diffusion into the electrolyte. I) Similar to (F), the addition of lactate in solution shows good sensitivity using the LOx/HRP functionalized gate. No shift in the baseline is observed when the gate is switched to the control.

To counter this toxicity, we devised an effective means of scavenging H_2_O_2_ by the addition of a peroxidase enzyme, in this case HRP or Cat, which converts the unreacted H_2_O_2_ into O_2_. By repeating the same functionalization steps as before, a second layer of PLL was added (Figure 1A iii), followed by the immobilization of HRP/Cat, again using EDC/s‐NHS activation chemistry (Figure 1A iv and **Figure** **3**A). QCM measurements showed, as previously with GOx, that when PLL was added as a spacer between the first enzyme (GOx/LOx) and the second enzyme (HRP) the frequency shift was higher, indicating that more enzyme remained on the surface (Figure S3E, Supporting Information). This highlights the fact that the second PLL layer helps to bind the secondary enzyme in the stack configuration. In order to investigate the H_2_O_2_ “blocking” ability of the HRP/Cat enzyme, a test was carried out, by introducing different concentrations of H_2_O_2_ into the electrolyte. The normalized current response (NR) was calculated for both control (only PEDOT:PSS:PVA and Pt‐NPs) and functionalized gates (with biostack layers). The same devices were measured before and after the functionalization with enzymes (Figure S5A, Supporting Information). The NR to H_2_O_2_ for a functionalized gate drops significantly compared to the control gate. This means that H_2_O_2_ introduced into the electrolyte, and diffusing toward the functionalized gate, is neutralized by the second enzyme, and does not reach the Pt catalytic surface (Figure S5B, Supporting Information). On the contrary, when different glucose concentrations were introduced into the electrolyte, the signal from the functionalized gate showed a typical sensing behavior, whereas signal modulation from the control gate was insignificant (Figure S5C,D, Supporting Information).

The role of HRP or Cat is to render the device nontoxic since production and consumption of H_2_O_2_ will remain local, at the gate electrode. This will allow integration of cells with the sensors and will additionally reduce interference when multiple sensors are desired within the same electrolyte. To test the ability of HRP to efficiently neutralize H_2_O_2_ while maintaining sensor performance however, a balance must be struck between the concentration ratio of the primary oxidase enzymes (GOx, LOx) and the second outer peroxidase enzymes (HRP, Cat), in order to maintain high sensitivity toward metabolite sensing while decreasing the toxicity of the out‐diffused H_2_O_2_ into the media. By tuning the concentration of the second enzyme with respect to the first enzyme, we effectively change the sensitivity of detection (Figure 3B,C). The NR for glucose detection is slightly shifted to higher concentrations upon utilization of larger concentration ratios of HRP, as expected. The initial GOx concentration added for immobilization was 1 mg mL^–1^, which gave a linear response at a concentration range between 50 × 10^−6^ and 1 × 10^−3^ m. Using a concentration ratio of HRP/GOx = 1/10 and HRP/GOx = 1/4 the NR shifts slightly. In the case of LOx, an initial concentration of 5 mg mL^–1^ was used, which gave a linear response in the concentration range of 3 × 10^−6^ –200 × 10^−6^ m. This higher concentration of LOx and the resulting sensitivity are desired in order to sense low concentrations of lactate produced by the cells. The addition of HRP affected more the changes in sensitivity for LOx functionalization more drastically than that of GOx (Figure 3B,C). A ratio that allowed good sensitivities as well as high biocompatibility was HRP /LOx = 1/50. This calibration must be performed empirically with respect to different activities of different enzymes; however, this is a useful optimization tool and shows the tunability of the system depending on the application and concentration range of interest. It should be noted that although HRP usually favors the oxidation of other substrates, previous work suggests that HRP can scavenge H_2_O_2_ and produce O_2_ in the absence of other reactants, mimicking the function of Cat.^[^ ^37^ ^]^

To further test both the diffusion of peroxide as well as the sensitivity of the device, an OECT was operated sequentially with one of two gates, either functionalized only with the primary redox enzyme (GOx or LOx; Figure 3D), or functionalized both with the primary redox enzyme as well as HRP (Figure 3G). In both cases, control gate electrodes with only Pt‐NPs were maintained in the same electrolyte in order to sense the H_2_O_2_ diffusing out from the functionalized gate. To monitor this out‐diffusion of H_2_O_2_, a constant gate voltage *V*_g_ = 0.4 V was applied to the functionalized gate, and the drain current (I_d_) was recorded (Figure 3E —GOx; Figure 3F —LOx). After a steady state I_d_ current baseline was observed, different concentrations of glucose (from 1 × 10^−6^–2.5 × 10^−3^ m) or lactate (from 1 × 10^−6^–250 × 10^−6^ m) were added, and between each addition step a waiting time was kept until the drain I_d_ reached a stable baseline. When switching to the nonfunctionalized control gate, the reaction of the decomposition of the diffused H_2_O_2_ in the electrolyte results in a new baseline; a difference, Δ*Ι*, of around 400 µA was observed for glucose and 200 µA for lactate. The difference is caused by H_2_O_2_ diffusing into the electrolyte that was produced by the functionalized gate as GOx or LOx turns over glucose or lactate, respectively. By performing the same measurements after the addition of HRP to the functionalization ([HRP]/[GOx] = 1/2) and ([HRP]/[LOx] = 1/10), we observe that when we switch to a control gate the *I*_d_ baseline reverts to initial levels (prior to addition of analytes), and no Δ*Ι* was observed, indicating that H_2_O_2_ has been effectively neutralized (Figure 3H —GOx; Figure 3I —LOx).

**Figure 4:**
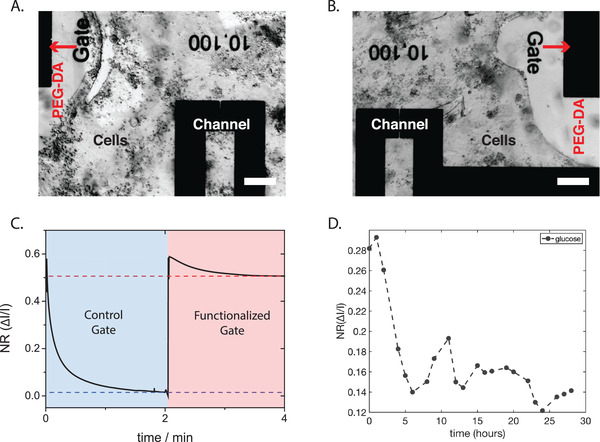
Detection of metabolites from live tissue cultured on biostack devices. A) Image of GOx/HRP device with PEG‐DA gel on gate and MDCK II cells grown around it. The toxicity of the sensor has been reduced as shown from the cells that are growing and proliferating around the gate. PEG‐DA gel on the gate prevents cells from growing directly on the electrode. Scale bar 200 µm B) Similar to (a), an image of an LOx/HRP device with gel on the gate and MDCK II cells grown on the neighboring substrate. Scale bar 200 µm. C) Normalized current response of GOx/HRP devices recorded from fully grown cells after fresh media is introduced. The blue highlighted region shows the time window for the response of a control gate, while the red area shows the time window for response of a functionalized gate. D) Normalized current response of continuous measurements of the glucose concentration in MDCK II cells.

To validate the nontoxicity of the biostack metabolite detection, the developed sensors were tested with live cells grown directly in the wells on the device. For these experiments with live cells, the gates were functionalized analogous to the protocol for characterization of the devices, using the biostack approach for glucose and lactate sensing. Furthermore, 2–5 wt% PEG‐DA in DI water, was drop‐cast on top of the gates and polymerized for 3 min under UV light. Owing to its bioinert properties, PEG prohibits cells from growing on top of the gate (masking the enzymes) and allows cellular metabolites to diffuse to the gate electrodes (**Figure** **4**A,B). MDCK II cells were seeded on the devices, forming a confluent layer in 4 d. OECT platforms have been shown to successfully measure the barrier properties of integrated epithelial cells.^[^ ^38^ ^]^ In our sensors, frequency dependent transconductance values verify the confluency of the cells and their barrier properties (Figure S6, Supporting Information). The cutoff frequency of the OECTs prior to seeding the cells is over 1000 Hz, whereas after cell confluency the cutoff frequency is below 100 Hz. These results are in agreement with previous work on OECT based impedance sensing and show that the cells exhibit barrier properties.^[^ ^39^ ^]^ After cell confluency, new media was added to the wells and glucose / lactate sensor measurements were carried out. In more detail, the applied *V*_g_ = 0.4 V was switched between the control gate (only Pt‐NPs) and the functionalized gate. The gate bias was applied to each gate until a steady state *I*_d_ baseline was observed (Figure S7, Supporting Information). In a GOx functionalized OECT, a large difference in the steady state *I*_d_ was observed between control and functionalized biased gates (≈50% of the control signal) (Figure 4C), which is in agreement with the calibration curves. In an LOx functionalized OECT, the *I*_d_ steady state difference was not observed when fresh media was added (Figure S8A, Supporting Information), however, a difference in *I*_d_ was observed after the cells were incubated for 12 h with media (Figure S8B, Supporting Information), indicating production of lactate from the cell metabolism. The method of pulsing between a control and a functionalized gate was used in order to discard any interference in the glucose or lactate signal that is common in complex media. The control gate provides a reference that does not change overtime.

As a proof of concept, continuous glucose measurements with cells were performed in a time window of 24 h, by interchanging the gate voltage between control and functionalized gate and recording the changes in I_d_ from a common OECT channel (Figure S9, Supporting Information). Our continuous measurement system was not capable of applying alternating gate biases exactly as used in previous characterization, in which the gate voltages were switched manually, but rather two voltages were applied at the OECT gates simultaneously. The effects of the measurement system, which are shown in Figure S10 in the Supporting Information, were taken into account to extract concentration changes during this measurement. Figure 4D shows the normalized I_d_ current response (NR) between functionalized and control gate over the course of 24 h, validating depletion of glucose by the cells.

## 3 Conclusion

In this report, we fabricate micrometer scale OECT based enzymatic sensors that can monitor relevant metabolites at the direct interface with the biological environment, without disrupting the integrity of the biological system. This is made feasible by functionalizing different enzymes in a stack that serve the desirable functions of sensing, increasing biocompatibility, and decreasing the interference. Electrodeposition of Pt‐NPs was performed in order to increase the catalytic activity of the gate for the turnover of H_2_O_2_. We functionalized the gate by covalently binding the biomolecules in a repeated order of PLL–GOx/LOx–PLL–HRP. We optimized the ratios of the enzymes in order to be able to sense in relevant metabolite concentrations but also limit the amount of diffused peroxide. The OECT based sensors were able to support the growth of MDCK II cells and monitor in situ the glucose consumption from their metabolism. The biostack approach aims to improve the biocompatibility of metabolite sensing platforms for applications that require metabolite monitoring at the interface with live cells and tissue. Examples of applications include metabolite sensing in brain tissue and drug toxicity screening.

## 4 Experimental Section

### Electrochemical Transistor Fabrication and Operation

The process, similar to that reported previously, ^[^ ^27^ ^]^included the deposition and patterning of gold, parylene C (PaC), and PEDOT:PSS:PVA. Source/drain contacts were patterned by a lift‐off process, using S1813 photoresist, exposed to UV light through a SUSS MBJ4 contact aligner, and developed using MF‐26 developer. 5 nm of chromium and 100 nm of gold were subsequently deposited using a metal evaporator, and metal lift‐off was carried out in acetone. Metal interconnects and pads were insulated by depositing 2 µm of PaC using an specialty coating systems (SCS) Labcoater 2, and a silane adhesion promoter. A dilute solution of industrial cleaner (Micro‐90) was subsequently spin coated to act as an antiadhesive for a second, sacrificial PaC (2 µm) film. Samples were subsequently patterned with a thick layer of AZ9260 photoresist (5 µm) and AZ developer (AZ Electronic Materials). The patterned areas were opened by reactive ion etching with an oxygen plasma using an Oxford 80 Plasmalab plus. PEDOT:PSS:PVA^[^ ^25^ ^]^ with GOPS (0.2% v/v) in solution was spin coated at 3000 rpm, and baked for 90 s in 100 °C. The second layer of parylene was peeled off with a subsequent rinsing in DI water and baking at 140 °C for 30 min.

### Pt‐NPs Deposition

Pt‐NPs were electrodeposited by reducing Pt ions. In more details, a solution containing chloroplatinic acid (H_2_PtCl_6_ 5 × 10^−3^ m from Sigma‐Aldrich) and sulfuric acid (H_2_SO_4_ 50 × 10^−3^ m from Sigma‐Aldrich) was used in a three‐electrode set up connected to a Metroohm potentiostat. As reference electrode an Ag/AgCl was used, as Counter electrode a Pt mesh, and as working electrode the planar gate of the OECT. Pulse Voltammetry was used with initial potential *V*_off_ = + 0.7 versus Ag/AgCl—where no reduction occurred for 60 s and final potential *V*_on_ = – 0.2 versus Ag/AgCl for 10 s where deposition of Pt occurred. Subsequently, the devices were soaked in Di water overnight for the removal of sulfuric acid, ethylene glycol, and dodecylbenzene sulfonic acid.

### 3‐Glycidoxypropyltrimethoxysilane (GOPS) Deposition

GOPS was deposited by CVD. A pelco mini desiccator with an aluminum base was placed on top of a hotplate and the temperature of the aluminum base was kept at 90 °C and was continuously monitored by a thermometer. The devices, as well as GOPS (200 µL) placed in an aluminum boat, were introduced to the desiccator. Subsequently, a continuous vacuum was applied by a pump connected to the desiccator. The deposition occurred for 1 h, then the samples were thoroughly rinsed with Di water for the elimination of weakly bonded GOPS, and a subsequent baking step took place for 30 min at the same temperature to facilitate subsequent crosslinking of silanol groups. During that step, glass cylindrical wells were placed on top of the samples, and a thin strip of uncured polydimethylsiloxane (PDMS) enabled a permanent sealing after curing.

### PLL Coating

PLL solution (1 µL drops of a 0.01 wt% from Sigma‐Aldrich) was drop‐casted on top of the gates and let for 3 h for reactions to take place. The device was in a high vapor pressure atmosphere in order to prevent evaporation of the droplets. Then the devices were thoroughly rinsed with PBS and DI water in order to remove the unbound PLL.

### Enzyme (GOx, LOx, HRP, Cat) Coating

Enzyme activation using EDC/NHS: GOx (10 mg mL^–1^ from Sigma‐Aldrich), LOx (kindly donated by Roche), and HRP (Sigma‐Aldrich) were separately mixed with EDC and sulfo‐NHS (50 × 10^−3^ m from Sigma‐Aldrich) in 2‐(N‐morpholino)ethanesulfonic acid (MES) buffer. The pH of the buffer was adjusted to pH 6 by addition of NaOH. The mixture left for 30 min at room temperature in order for the carboxylic acid to be activated to s‐NHS ester. Afterward, PBS 1X was added to the mixture in order to bring the pH to 7.4 and the desired final concentration of each enzyme. Finally, 2 µL of the activated enzyme was drop‐casted on top of each gate and let for 3 h for reactions to take place (s‐NHS group of the enzyme to bind to the NH2 group of the PLL) in room temperature. The device was in a high vapor pressure atmosphere in order to prevent evaporation of the droplets. The samples were rinsed three times with PBS 10X and DI water. The same process continued with alternation of enzyme layer of interest and PLL, in order to create the biostack. All the steps were performed in sterile conditions. Catalase was used in some of the experiments as an outmost enzyme layer instead of HRP as in Figures S3 and S5 in the Supporting Information.

### PEG‐DA Hydrogel

PEG‐DA (*M*_W_ ≈ 6000 10 wt%) in DI water was sonicated until a clear solution was obtained. Subsequently, 2‐hydroxy‐2‐methylpropiophenone (2 wt% from Sigma‐Aldrich) photoinitiator was introduced to the solution and mixed extensively. The hydrogel (1 µl) was drop‐casted on top of the gate and photopolymerized with a UV ≈ 365 nm led array for 3 min (the time was kept short, in order to retain the structure and functionality of the enzyme). Finally, the devices were rinsed thoroughly with PBS 1X and DI water, and stored with PBS 1X in the fridge.

### Cell Culture

Epithelial MDCK II cells (kindly donated by Dr. Frédéric Luton, Institute of Cellular and Molecular Pharmacology, Valbonne, France) were routinely maintained at 37 °C in a humidified atmosphere of 5% CO_2_ in complete Dulbecco's Modified Eagle Medium (DMEM) (cDMEM) media (Dulbecco's Modified Eagle Medium, fetal bovine serum (10%), and Pen/Strep 5000 [U mL^–1^] penicillin–5000 [µg mL^–1^] streptomycin). For all experiments, cells were incubated at an initial density of 6×10^4^ cells/transwell insert (0.4 µm pore size, and area of 0.33 cm^2^) and incubated until they reached confluence.

### Device Operation

The measurements were performed using a National Instruments PXIe‐1062Q system. The channel of the OECT was biased using one channel of a source‐measurement unit NI PXIe‐4145. The gate voltage was applied using an NI PXI‐6289 modular instrument. In the case of the IV‐characteristics and transconductance characterization, the drain current was measured using the system measurement unit. The same channel was used for the bias. Concerning the time response characterization of the OECT, one NI‐PXI‐4071 digital multimeter measured drain current, and a channel of the NI PXI‐6289 equipment measured gate voltage. All the measurements were triggered through the built‐in PXI architecture. For the continuous glucose monitoring in MDCK II cells, two voltages were applied simultaneously for every measurement, meaning that, when the voltage in the functionalized gate was positive (*V* = 0.4 V) the voltage at the control gate was at *V* = 0 V, and not in open circuit. This lowers the effective gate voltage and thus the dedoping at the channel, lowering the normalized current response and shifting the sensitivity toward catalysis of H_2_O_2_ (Figure S10, Supporting Information).

### QCM Measurements

Samples were prepared as follows. QCM crystals, if not new, were cleaned with Piranha solution, rinsed thoroughly with di‐water, and dried with N_2_. PEDOT:PSS:PVA was spin coated (3000 rpm for 30 s) on QCM gold quartz crystals. Oxygen plasma (Oxford instruments, Power: 100 W, O_2_ 60 sccm, Pressure 100, time 120 s) was done to improve film formation. GOPS was deposited by CVD as mentioned above. The crystals were inserted in the QCM‐D (Biolin Scientific), and the solutions with PLL and enzymes were flashed sequentially to create the stack configuration. Once the frequency was shifting, the flow was stopped in order for reactions to occur, and between each solution of interest. PBS 1X was inserted as washing step, until the frequency was stabilized. Each frequency curve was then extracted and normalized to the PBS background.

### Statistical Analysis

All data and statistical analysis was performed using MATLAB software from MathWorks. For the calibration curves in Figure 3B,C, three different samples (*n* = 3) for each condition and two time series for each sample n were performed. The average and standard deviation was taken for each condition. The calibration curves in Figure S5 in the Supporting Information were fitted by using the Langmuir adsorption isotherm, since the catalysis of H_2_O_2_ on the Pt‐NPs is a surface reaction^[^ ^40^ ^]^

(1) $$\mathrm{NR}=\frac{K*C_{h}}{1+K*C_{h}}$$

where *K* is the rate constant and *C*_h_ the concentration of H_2_O_2_. Figure S6 in the Supporting Information, the transconductance versus the frequency was fitted with sinusoidal model by using a custom MATLAB function.

## Conflict of Interest

The authors declare no conflict of interest.

## Supporting information

Supporting Information

## Data Availability

The data that support the findings of this study are available from the corresponding author upon reasonable request.
