# Supplementary material for: Biostack: Nontoxic Metabolite Detection from Live Tissue
Source: Adv Sci (Weinh). 2021 Nov 5;9(2):2101711. doi: 10.1002/advs.202101711 (PMC8805579; doi:10.1002/advs.202101711)
Supplement: Supplementary file 1 — Supporting Information [file ADVS-9-2101711-s001.pdf]

© 2021 Wiley-VCH GmbH

ADVANCED SCIENCE
Open Access

Supporting Information

for *Adv. Sci.*, DOI: 10.1002/advs.202101711

Bio-stack: Non-toxic metabolite detection from live tissue

Xenofon Strakosas, [1]*† *Mary. J. Donahue,* [1]† *Adel. Hama, Marcel. Braendlein*[4], *Miriam. Huerta*[2], *Daniel. T. Simon,* [1] *Magnus. Berggren,* [1] *George. G. Malliaras*[5], *R. M. Owens*[6]*

Supporting Information

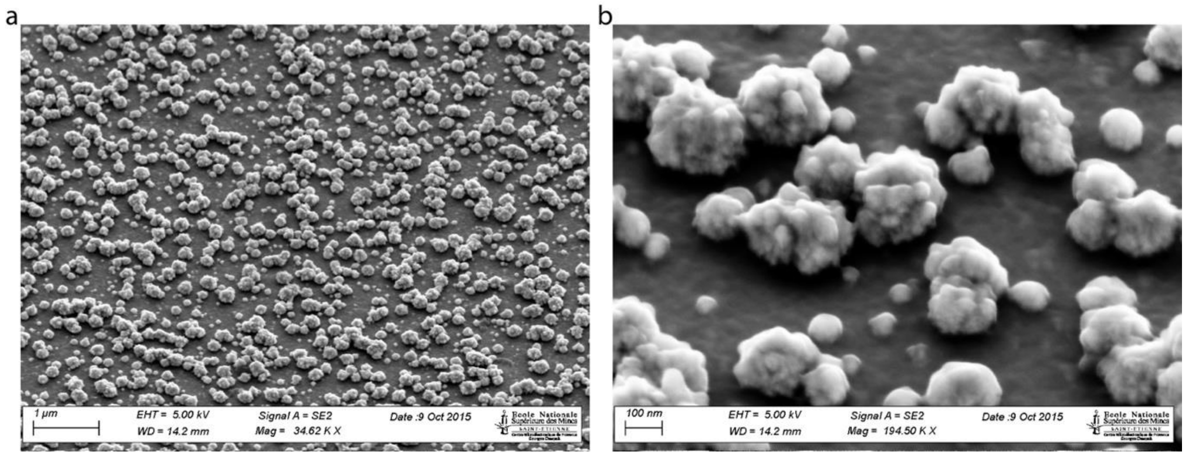

Figure S1. SEM image of gate of OECT after Pt NP deposition. a and b images show the ‘popcorn’ like appearance of the Pt-NP deposited onto the PEDOT:PSS.

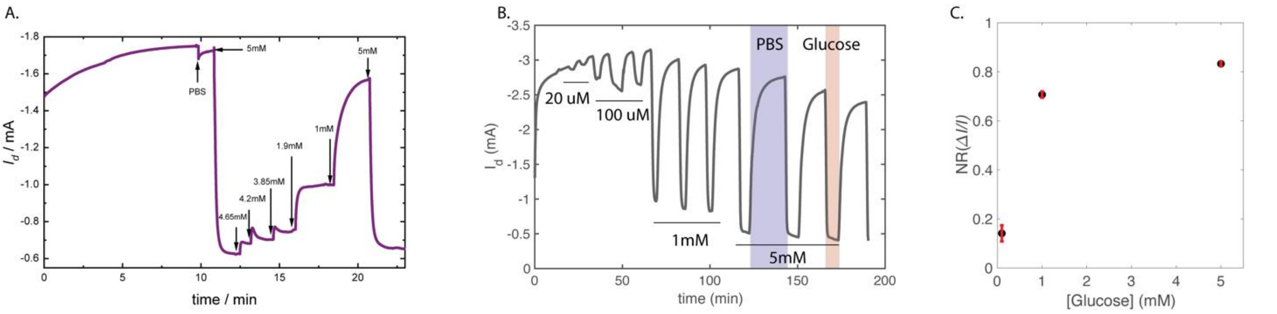

Figure S2. Current response upon addition of various concentrations of glucose A. current response over time starting with high concentration of glucose and stepwise dilution. B. Id current response upon addition of various concentrations of glucose with washing steps with PBS in between. A constant gate voltage $V_g = 0.4$ V is applied to the OECT. C. Mean normalized response (NR) from the currents in Figure S2B showing that the modulation of the current is not affected from the drifting of the baseline but only from the concentration of the analyte.

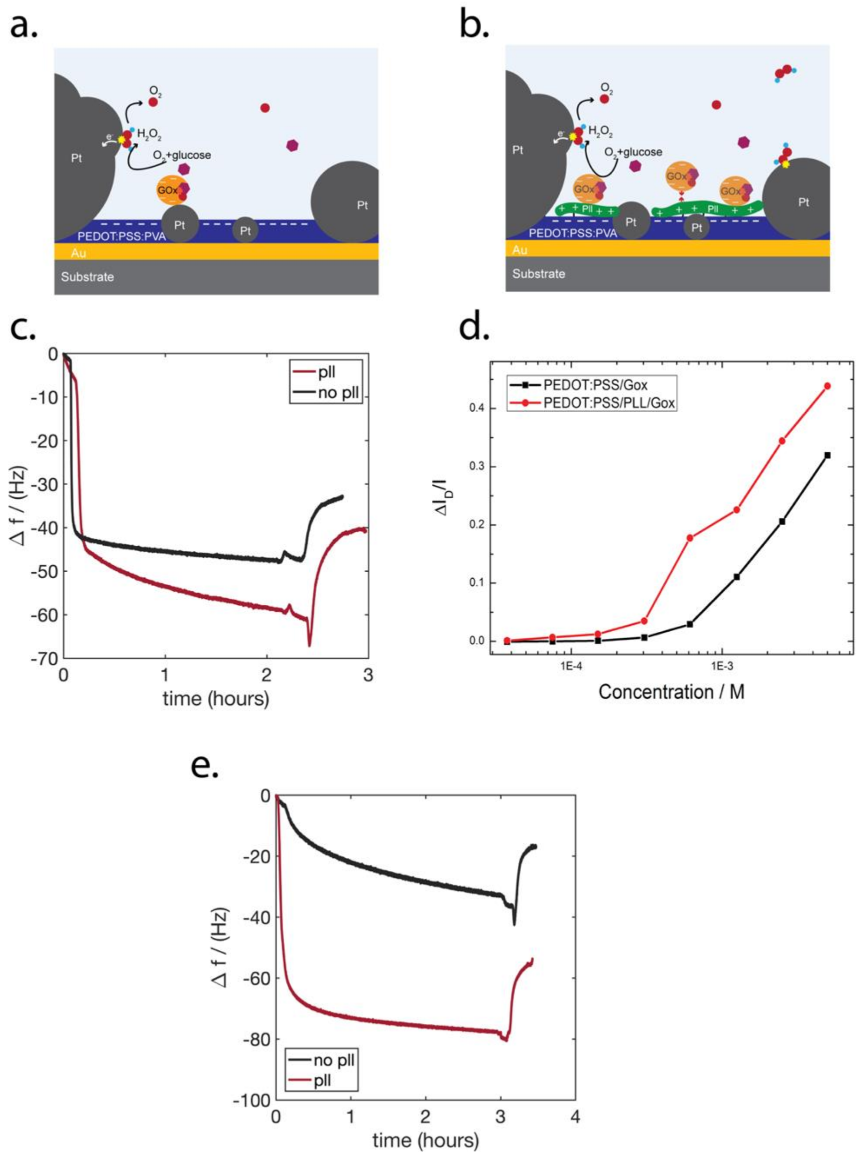

Figure S3. Effect of PLL on metabolite detection using the Biostack device. a, b) Schematic showing immobilization of GOx without and with prior PLL addition. c) QCM measurements for immobilizing GOx onto the PEDOT:PSS:PVA. The change in the frequency, which is proportional to the effective mass immobilized on PEDOT:PSS:PVA/GOPS in the absence (black) and presence of PLL (red). After 2.5 hours a cleaning step with PBS, DI water removes the adsorbed GOx and GOx existing in solution, shifting the frequency. d) Normalized I_d current response versus glucose concentration for sensors when GOx was immobilized on PLL (red curve) and when GOx was immobilized on PEDOT:PSS:PVA without PLL e) QCM measurements for immobilizing Catalase onto the GOx in order to form the enzymatic stack. The change in the frequency, in the absence of a

second PLL layer on top of GOx (black) and presence of second PLL layer on top of GOx (red). After 2.5 hours a cleaning step with PBS, DI water removes the adsorbed GOx and GOx existing in solution, shifting the frequency to lower values.

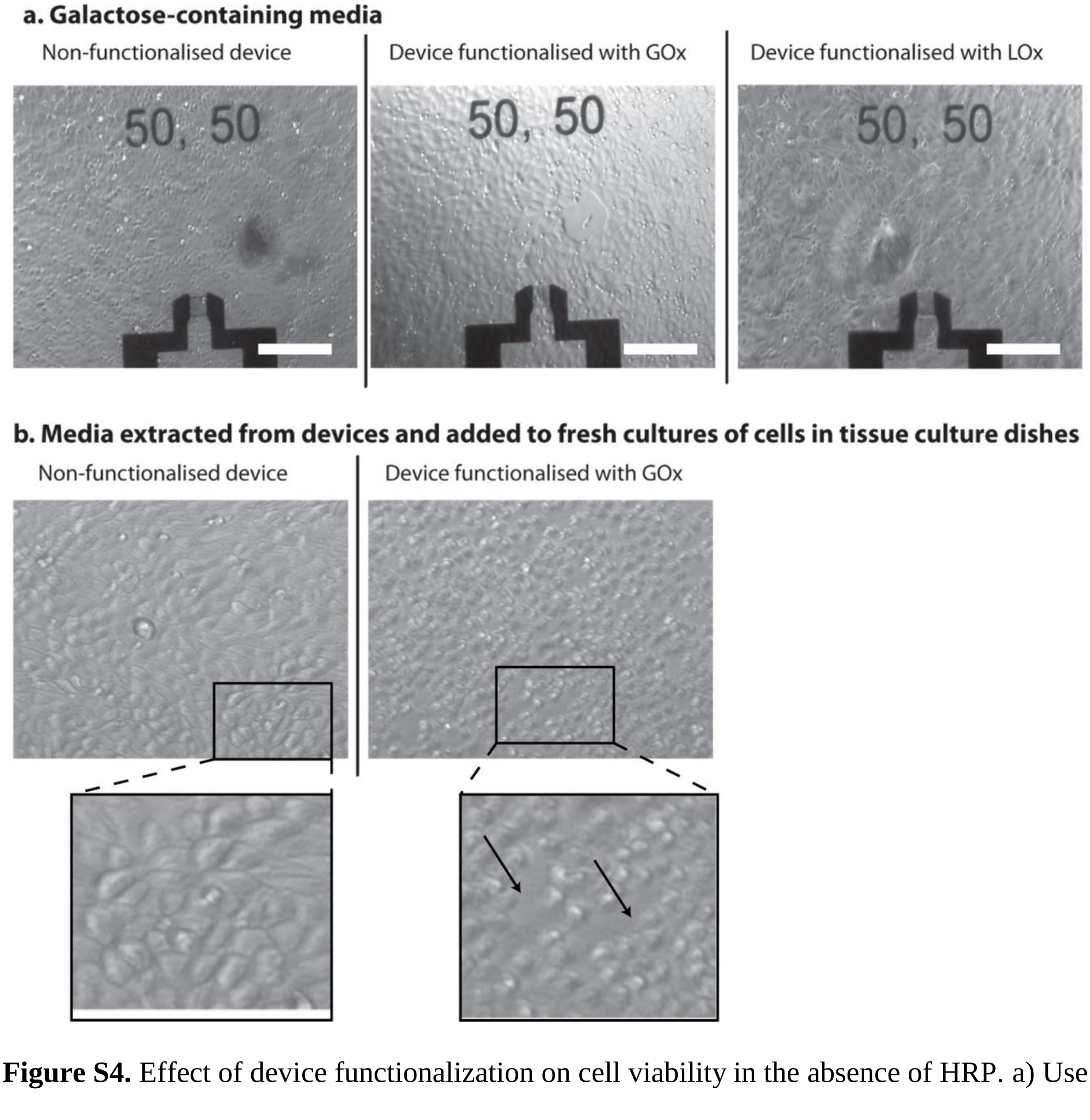

Figure S4. Effect of device functionalization on cell viability in the absence of HRP. a) Use of an alternative carbon source (galactose) shows improved viability of GOx functionalized device, since there is no substrate for the GOx to turnover, and therefore no generation of peroxide. Scale bars 200 μm. b) Media extracted from devices either non-functionalized or functionalized with GOx was

added to fresh MDCK II cells grown in tissue culture dishes. Aberrant morphology of the cells with media from the GOx device is apparent, whereas the media from the non-functionalized devices does not appear to adversely affect the cells. Zooming in the images show the elongated shape of healthy cells (left), and round shape of non-healthy cell (right) as well as empty areas (black arrows).

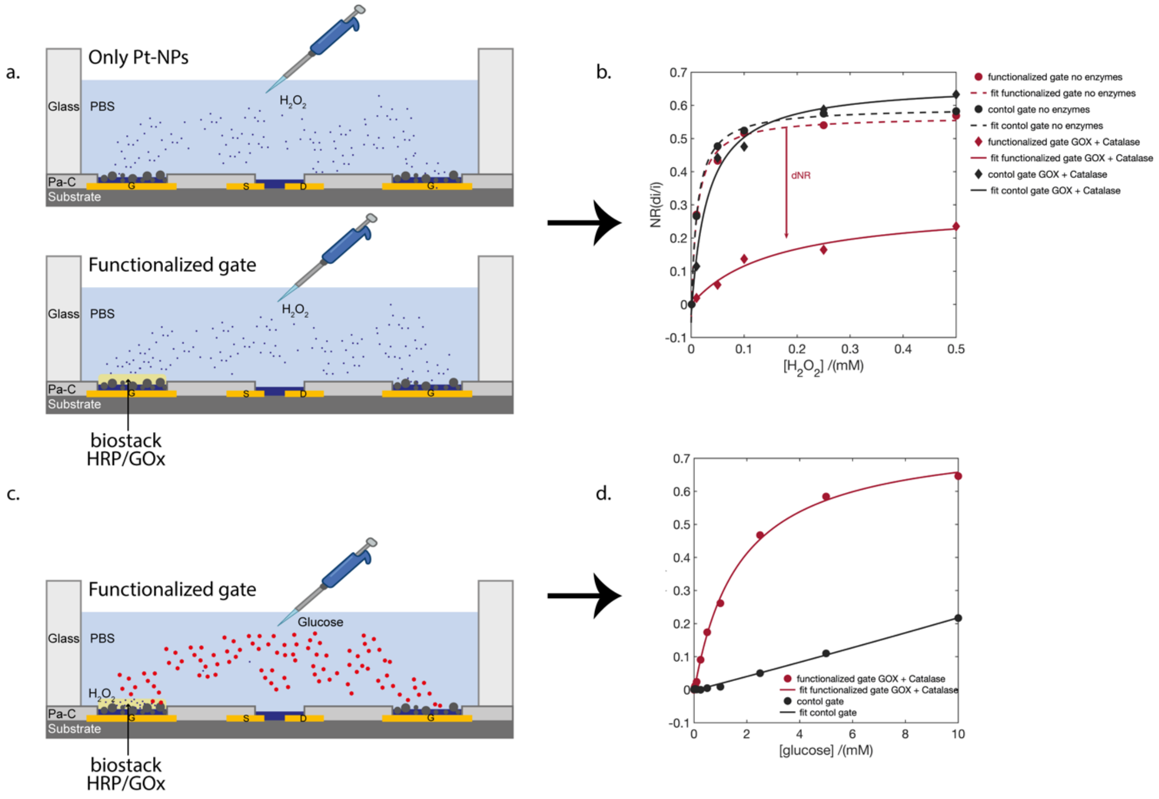

Figure S5. Sensing of diffused H_2O_2 in the electrolyte. a) schematic showing the experimental set up, in which we add different concentrations of H_2O_2 in PBS while sensing with the OECT for the control and functionalized gate prior to the biostack functionalization (TOP schematic). Bottom schematic, repeating the process after addition of biostack to the functionalized gate. b) Normalized current response (NR = dI/I_0) for OECTs upon addition of H2O2 in solution, prior (black, and red circles) and after enzyme functionalization (black and red diamonds) for both functionalized and control gates. The normalized response for a functionalized gate has decreased towards the catalysis of external H_2O_2, whereas the control gate bovine serum albumin (BSA) immobilization does not show big changes. c) schematic showing the experimental set up, in which different concentrations

of glucose were added in solution while sensing with the functionalized and control gate. d) Normalized current response for OECTs upon addition of glucose in solution for a functionalized gate (red circles) and a control gate (black circles). For high glucose concentrations (e.g., 10 mM), H_2O_2 produced at the functionalized gate can diffuse to the solution and be sensed from the control gate.

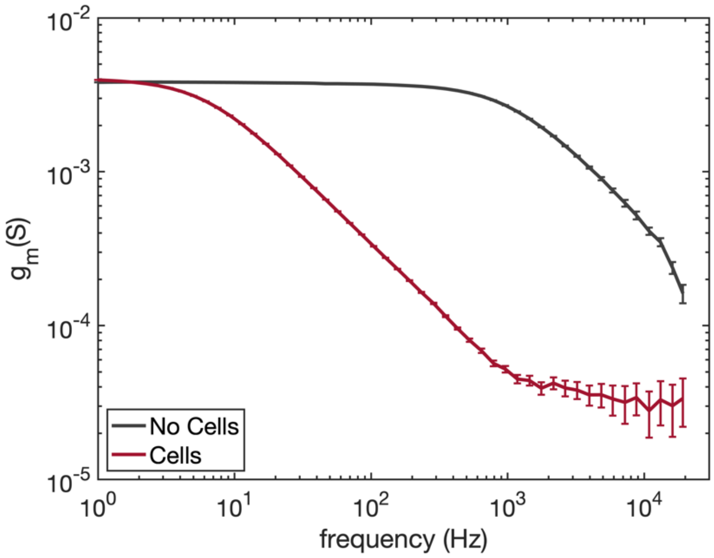

Figure S6. Frequency dependence of transconductance of an OECT without cells (black line) and with (red line) MDCK cells cultured on top of OECT sensors.

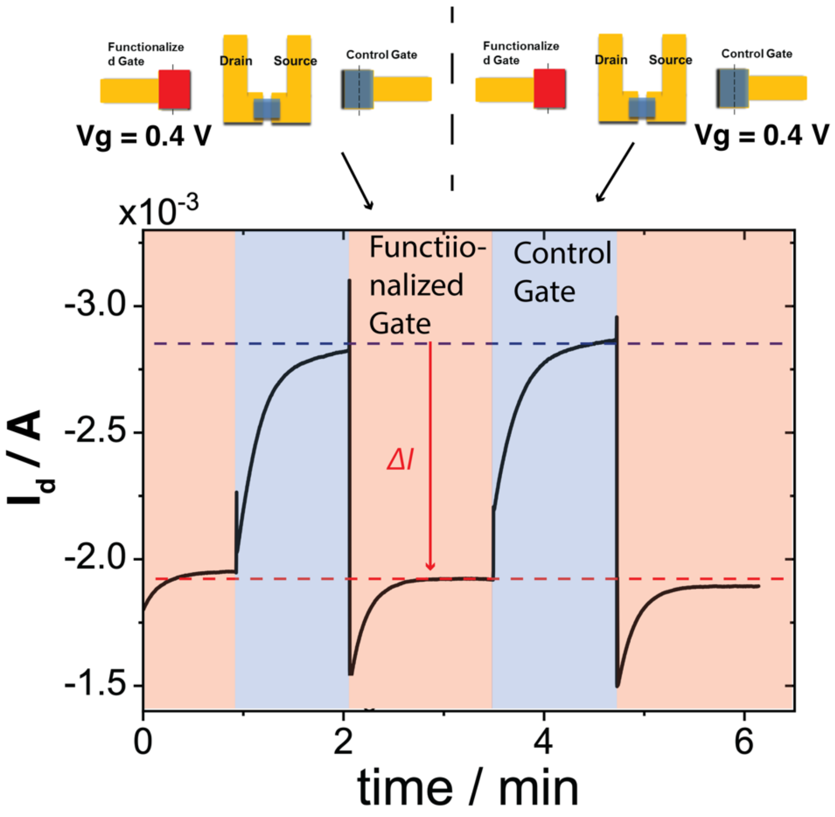

Figure S7. I_d current response for glucose measurements in the presence of cells from a functionalized and a control gate. a representative I_d current response of an OECT channel, between control and functionalized with GOx:HRP gate in the presence of confluent cells in fresh media with 5 mM glucose concentration. A sequential switching of a gate voltage (V_g = 0.4 V) is applied between control and functionalized gate. The enzymatic reaction at the functionalized gate and further the catalysis of H_2O_2 from the Pt-NPs result in de-doping at the channel, thus lower I_d.

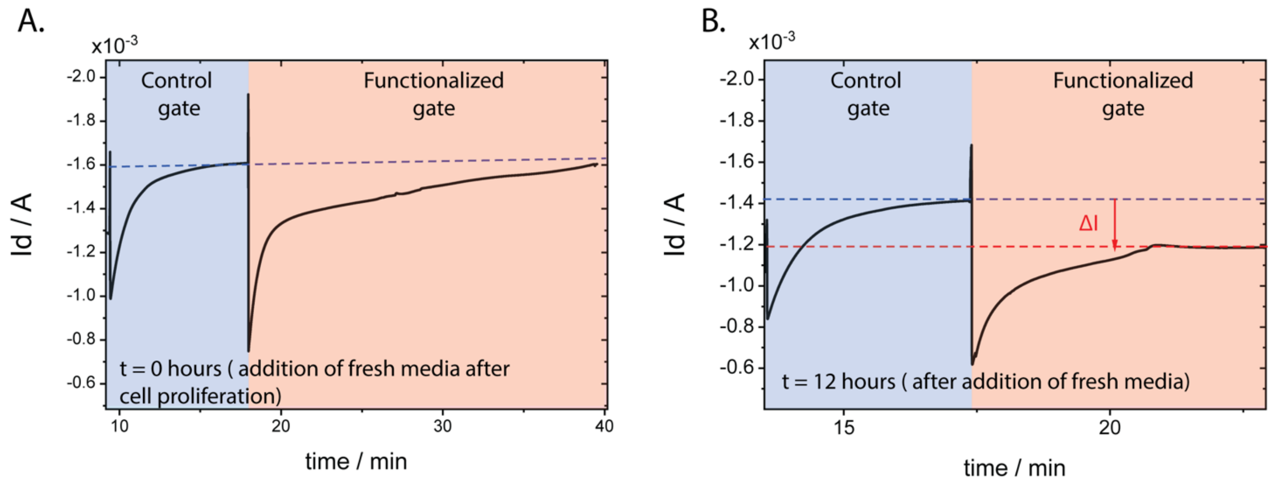

Figure S8. I_d current response for lactate measurements in the presence of cells from a functionalized and a control gate. a) Similar to Figure S7 a representative I_d current response of an OECT channel, between control and functionalized with LOx:HRP gate in the presence of confluent cells immediately after cell media was replaced with fresh media. b) a representative I_d current response of an OECT channel, between control and functionalized with LOx:HRP gate in the presence of confluent cells 12 hours after incubation of cells with media.

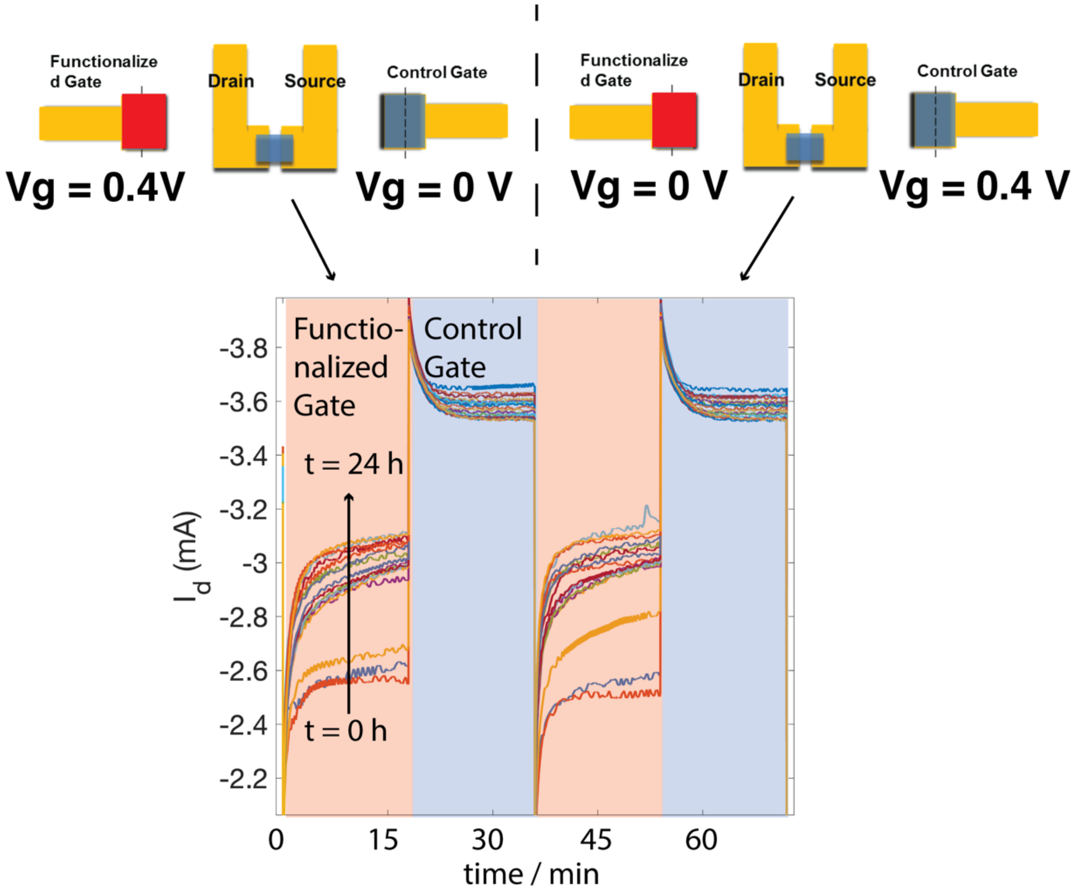

Figure S9: Id current response from continuous glucose measurements with OECTs based sensors. I_d response for continuous glucose monitoring, are overlapped to show the difference in the current magnitude overtime. A Vg = 0.4 V and a Vg = 0 V was applied alternatively from a functionalized with GOx:HRP and control gate. The magnitude of the I_d current, when V_g = 0.4V at the functionalized gate (orange band), increases overtime, while the Id current, when V_g = 0.4V at the

control gate shows no significant changes. This shows consumption of glucose that occurs from the metabolism of cells overtime.

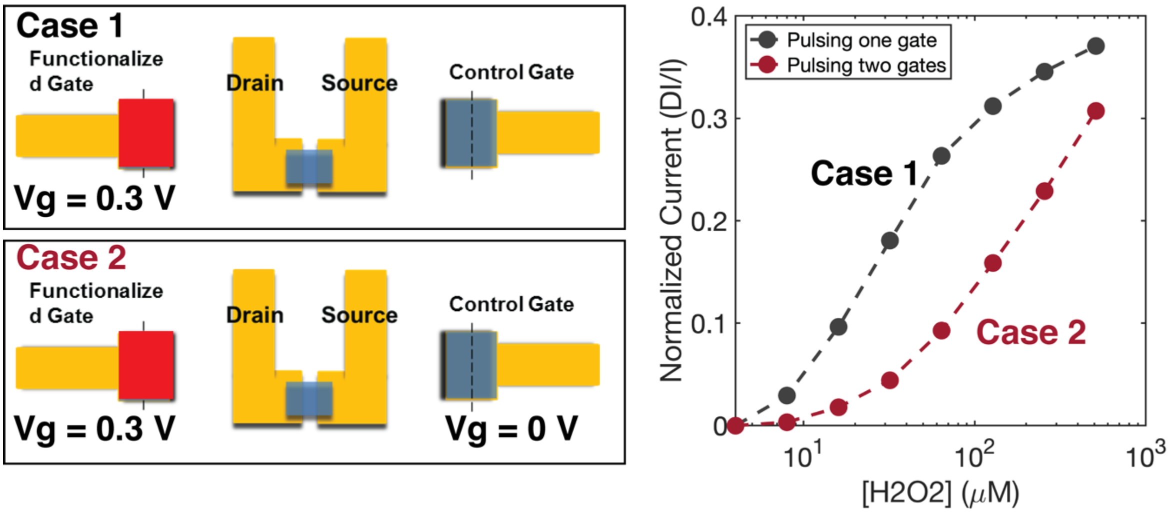

Figure S10: Change in the sensitivity upon a two-gate voltage application. The measurement system was not capable of having a floating gate and was applying 0 V on the second gate. Calibration curves from Pt-NPs functionalized gate towards H_2O_2. When only the functionalized gate is applying a constant voltage V_g = 0.3 V, the changes in current towards addition of H_2O_2 occur for lower concentrations of H_2O_2. When both gates are applying at the same time with V_g = 0.3 V for the functionalized gate, and V_g = 0V for the control gate, the changes in current towards addition of H_2O_2 occurs in higher concentrations. That is to say, the sensitivity of the sensor drops when two gates are being operated at the same time.
